# Supplementary material for: The Measurement of Love: Psychometric Properties and Preliminary Findings of the Short Love Scale (SLS-12) in a Polish Sample
Source: Int J Environ Res Public Health. 2022 Oct 14;19(20):13269. doi: 10.3390/ijerph192013269 (PMC9603604; doi:10.3390/ijerph192013269)
Supplement: Supplementary file 1 [file ijerph-19-13269-s001.zip › ijerph-1914621-supplementary.pdf]

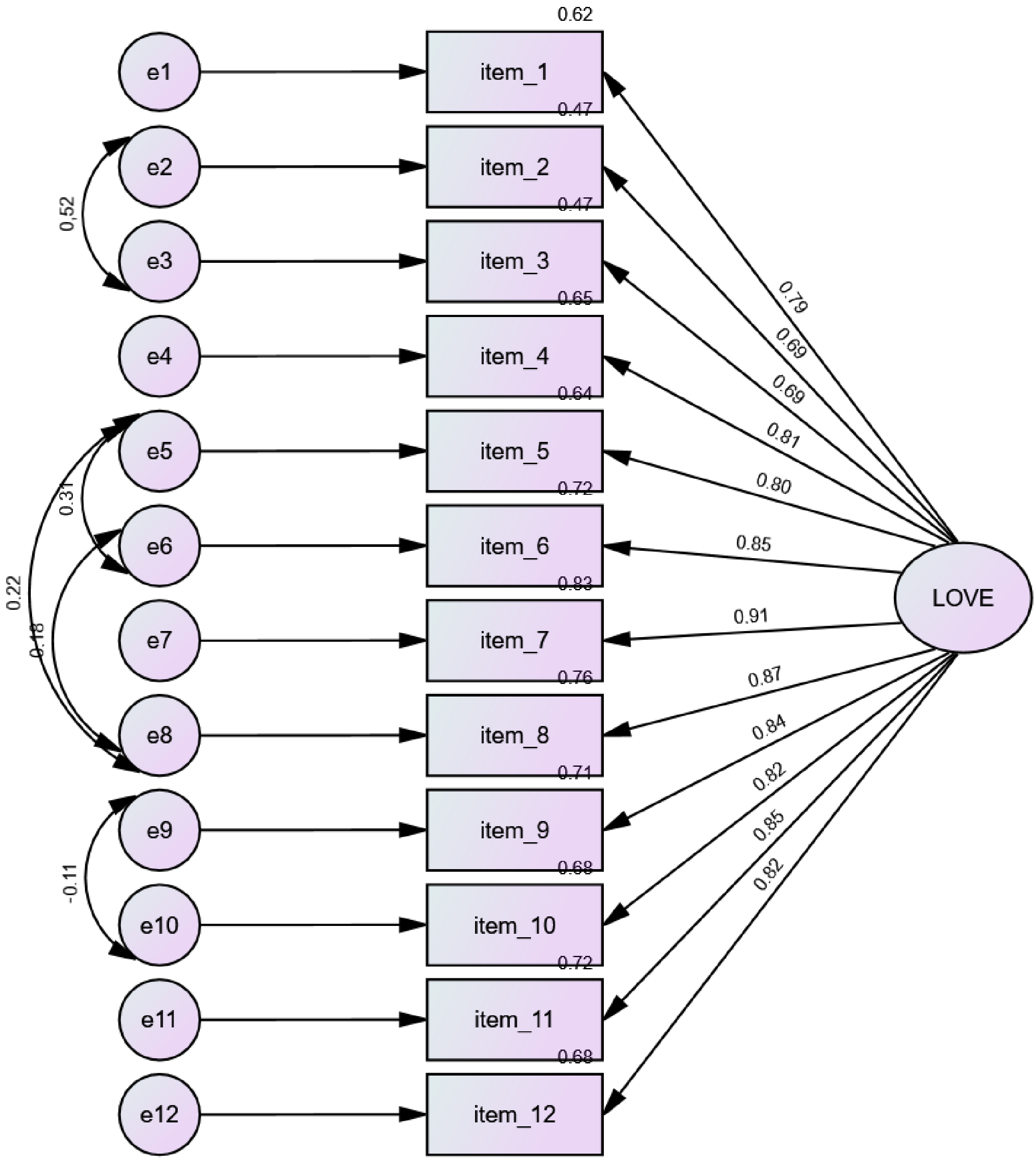

Figure S1. One-factor SLS-12 model based on EFA solution (n=470)

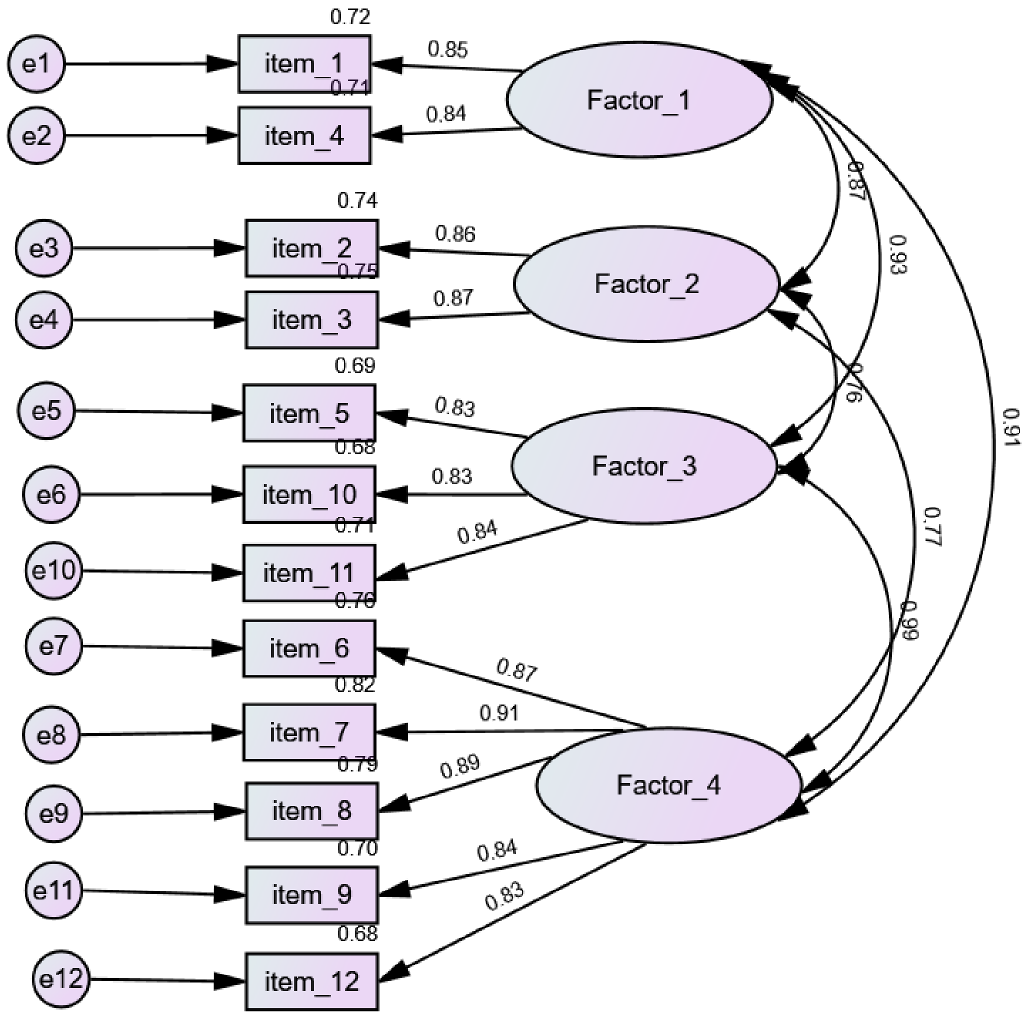

Figure S2. Four-factor SLS-12 model based on EFA solution (n=470)

Table S1. Exploratory factor analysis (EFA) of the Short Love Scale (SLS-12; n = 471).

Variables	Factor 1
item_1	0.668
item_2	0.556
item_3	0.540
item_4	0.689
item_5	0.679
item_6	0.724
item_7	0.809
item_8	0.786
item_9	0.733
item_10	0.780
item_11	0.727
item_12	0.761

Promax with Kaiser normalization; factor loadings below 0.40 are not presented

Table S2. Exploratory factor analysis (EFA) of the Short Love Scale (SLS-12; n = 471).

Variables	Factor 1	Factor 2	Factor 3
item_1		0.804	
item_2	0.835		
item_3	0.895		
item_4		0.675	
item_5			0.640
item_6			0.756
item_7			0.614
item_8			0.885
item_9			0.922
item_10			0.638
item_11			0.490
item_12			0.778

Promax with Kaiser normalization; factor loadings below 0.40 are not presented.

Table S3. Exploratory factor analysis (EFA) of the Short Love Scale (SLS-12; n = 471).

**Variables**	**Factor 1**	**Factor 2**	**Factor 3**	**Factor 4**
item_1	0.638			
item_2		0.850		
item_3		0.918		
item_4	0.765			
item_5			0.851	
item_6				0.555
item_7				0.481
item_8				0.647
item_9				0.868
item_10			0.484	
item_11			0.458	
item_12				0.757

Promax with Kaiser normalization; factor loadings below 0.40 are not presented
